# Taxonomic note on *Parnassia* (Celastraceae): the identity of *P. nubicola*

**DOI:** 10.3897/phytokeys.154.54042

**Published:** 2020-08-04

**Authors:** Yonghong Ma, Hanlu Zhou, Yumin Shu

**Affiliations:** 1 College of Life Science, China West Normal University, Nanchong 637002, Sichuan, China China West Normal University Nanchong China; 2 Key Laboratory of Southwest China Wildlife Resource Conservation (Ministry of Education), Nanchong 637002, Sichuan, China China West Normal University Nanchong China

**Keywords:** *Flora Iranica*, Himalaya, morphology, revision, staminode

## Abstract

Based on examination of herbarium specimens (including types) and living plants, as well as analysis of protologues and distributions, *Parnassia tibetana*, P. nubicola subsp. occidentalis, and P. nubicola var. nana are reduced to synonyms of *P. nubicola*.

## Introduction

*Parnassia*Linnaeus (1753: 273), a morphologically distinguishable genus in the Celastraceae (APG IV 2016, Ball 2016), is distributed in the Northern Hemisphere, predominantly in temperate regions. The Pan-Himalaya region is the center of this genus’ distribution as well as diversification (Phillips 1982, Ku and Hultgard 2001, Simmons 2004, Wu et al. 2004). All species of *Parnassia* are glabrous and rosulate perennial herbs, with solitary flowers borne on scapes. Flowers of all *Parnassia* species are pentamerous, actinomorphic or weakly zygomorphic, especially with the antipetalous staminodes. Morphologicaly, *Parnassia* is a rather homogeneous genus.

*Parnassia nubicola* Wall. ex Royle (1835: 227) is widely distributed in the mountainous regions of Himalayas; it is highly variable in some of its morphological characters, especially some quantitative characters such as leaf blades length, petals length, and plant height (Schönbeck-Temesy 1966, Grierson 1987, Ku and Hultgard 2001).

In this study, herbarium sheets from BJFC, BM, BNU, E, GZU, K, KUN, L, NY, PE, QTPMB, U, and W (herbarium acronyms follow Thiers 2020, continuously updated) herbaria and two populations of living plants (voucher specimens were deposited in PE) were examined.

The typification of *Parnassia nubicola* has been discussed (Shu et al. 2017). This species was described based on *Wallich Cat. n. 1246*, without specifying the herbarium in which the specimen was deposited. However, specimens with this collection number were either obtained by Wallich from Gossain Than (*Wallich Cat. n. 1246a*), or by Blinkworth from Kumaon (*Wallich Cat. n. 1246b*). In addition, some individuals with fimbriate petals in these collections are more accurately identified as *P. wightiana* Wall. ex Wight et Arnott (1834: 35). A specimen of *Wall. 1246a* in W has been designated as the lectotype of *P. nubicola* (Schönbeck-Temesy 1966), and all the other duplicate specimens of *Wall. Cat. n. 1246a* are isolectotypes, except for the individuals with fimbriate petals that were determined to be *P. wightiana*.

Based on *Wall. Cat. n. 1246b* collected from Kumaon, Drude (1875) named the individuals with fimbriate petals and cordate leaves (actually *Parnassia wightiana*) as the new variety P. nubicola var. cordataDrude (1875: 316). This has been previously discussed by Shu et al. (2017).

Parnassia nubicola subsp. occidentalisSchönbeck-Temesy (1966: 2) was described with cordate leaf blades (vs. ovate-elliptic to ovate in P. nubicola subsp. nubicola), lanceolate or lanceolate-oblong sepals (vs. ovate to ovate-lanceolate), punctate petals (vs. not punctate), and entire or erose petal margins (vs. erose to short-fimbriate). Ku described a new variety, P. nubicola var. nana T.C. Ku (1991: 82), based on the size of the leaf blade (ca. 2×1.5 cm vs. 2.5–7.5 × 2–3.8 cm in P. nubicola var. nubicola) and plant height (5–13 cm vs. 13–40 cm). P. nubicola subsp. occidentalis and P. nubicola var. nana were recognized by a combination of these variable characters.

Our analysis of specimens from herbarium collections, including types of *P. nubicola*, P. nubicola subsp. occidentalis, and P. nubicola var. nana, suggested that these highly variable characters are continuous, and cannot be used to reliably separate these taxa (Table 1). Leaf blades from the holotype of P. nubicola subsp. occidentalis are ovate, rather than cordate. Extensive field observations and examination of herbarium specimens showed that petal punctation is always clearly visible in old herbarium specimens, whereas it is rarely observable in the living individuals or newly collected specimens. This phenomenon was also observed in *Parnassia* by Suksathan (2009), petal punctation speculated to be an artifact of preservation due to dessication.

**Table 1.** Comparison of key morphological characters of *Parnassia nubicola*, P. nubicola subsp. occidentalis, P. nubicola var. nana, and *P. tibetana*.

Characters	P. nubicola subsp. nubicola*	P. nubicola subsp. occidentalis**	P. nubicola var. nana**	P. tibetana*
height	10–40 cm	15–33 cm	5–20 cm	12–20 cm
basal leave shape and size	ovate or ovate-oblong, 1–9.5 × 0.8–5.2 cm	ovate-oblong or ovate, 1.5–3.5 × 0.7–2 cm	ovate, 1.2–2.5 × 0.8–2.5 cm	ovate-oblong, 1.5–2.3 × 1–1.4 cm
sepal shape and size	ovate-oblong or ovate-lanceolate, 5–8 × 2–3 mm	ovate-lanceolate, ca. 5 × 3 mm	ovate-lanceolate, 4–6 × 2–3 mm	ovate, ca. 6 × 3 mm
petal shape and size	ovate, oblong or obovate, 12–16 × 8–10 mm, margins entire, erose or short fimbriate at base	obovate, 12–15 × 5–7 mm, margins subentire	obovate, 12–13 × 7 mm, margins entire or erose at base	obovate or oblong, 8–10 × 5–6 mm, margins erose at base
staminode shape	3-lobed shallowly to half its length, apex acute or rounded	3-lobed shallowly, apex rounded	3-lobed for 1/4–1/2 its length, apex acute or rounded	3-lobed for 1/3 its length, apex rounded

*Description based on the Flora of China (Ku and Hultgard 2001), specimens from BJFC, BM, BNU, E, GZU, K, KUN, L, NY, PE, QTPMB, U, W, and observations of wild individuals from Yunnan and Tibet. **Description based primarily on reexamination of holotypes as well as the original publication.

**Figure 1.**
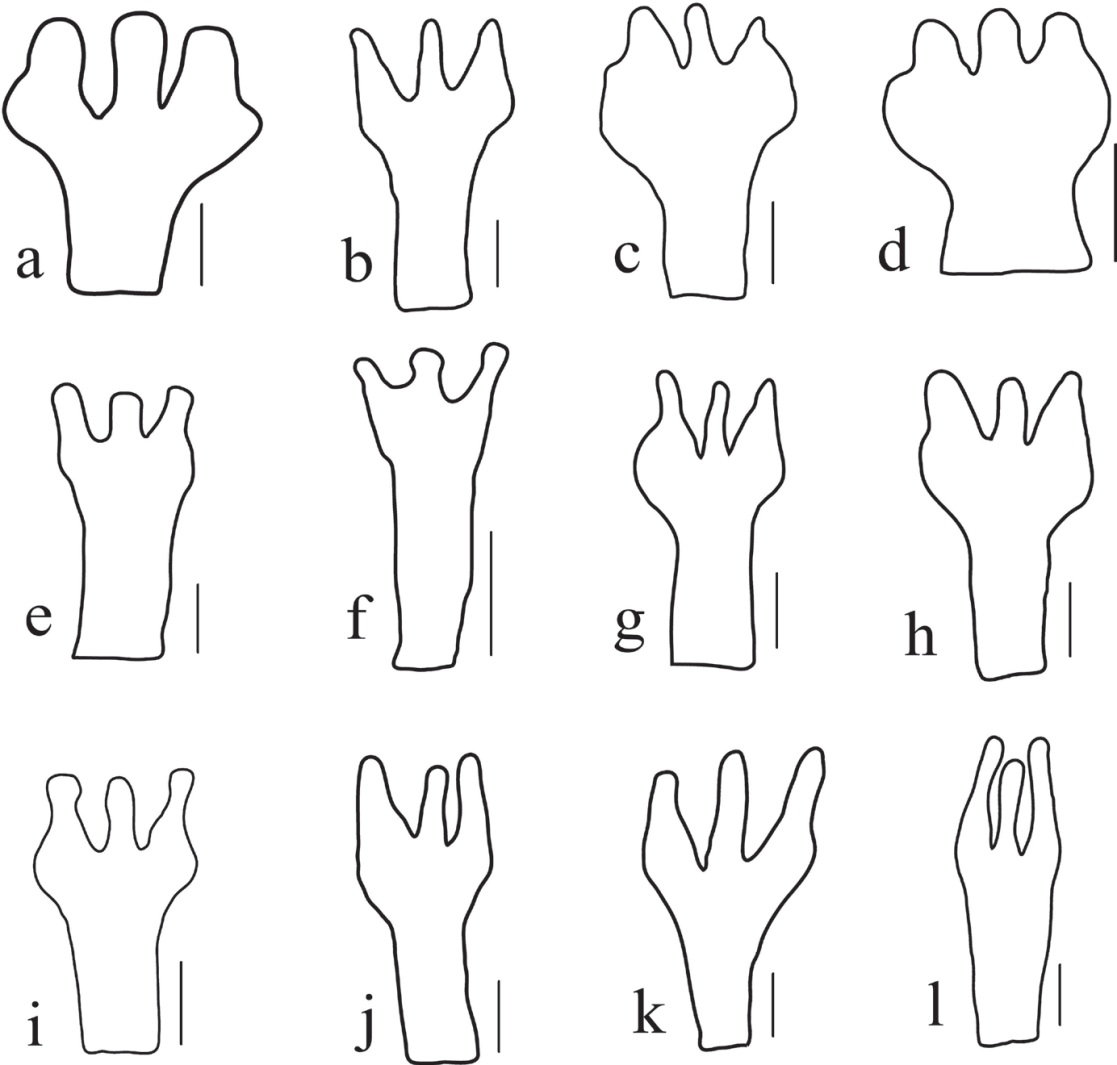
Staminodes variation of *P. tibetana* (**A**), P. nubicola var. nana (**B–D**) and P. nubicola var. nubicola (**E–L**). **A** delineated from *s.n. 716* (PE01863973) **B–D** delineated from *Hengduan Exp. 3734* (PE00866288 and PE00866291) **E–L** delineated from *Tibet herbs Exp. 1496* (PE00866275), *Plateau Exp. 14709* (PE00866299), *s.n. 6550* (PE01982606), and *Wang Qiwu 65371* (PE00866286). Scale bars: 1 mm. Illustrated by Shu Yumin.

*Parnassia tibetana* Z.P. Jien ex T.C. Ku (1987: 38), described on the basis of a single collection from Tibet, China, is found to be conspecific with *P. nubicola*. According to the original description, *P. tibetana* is morphologically similar to *P. nubicola*. The significant differences are that *P. tibetana* has yellow petals, shortly linear staminode lobes, which are rounded or truncate at the apex; in contrast, *P. nubicola* has white petals, and lanceolate or ovate-lanceolate staminode lobes, with the apex acute or rounded. We have examined the holotype of *P. tibetana* in PE and an isotype from N. When we compared these to various specimens of *P. nubicola*, as well as to wild individuals in Yunnan and Tibet, we found them to be part of the broader distribution of natural variation. In the flowering stage, *P. nubicola* petals are white (sometimes with yellow at the base), but the petals turn yellowish when the plant is in fruit, or when preserved as herbarium specimens. The delineated staminodes from some specimens, including type materials of *P. tibetana* and P. nubicola var. nana, show that staminode shape is highly variable (Fig. 1). The variation of staminodes in *P. mysorensis* Heyne ex Wight et Arnott (1834: 35), *P. wightiana*, and *P. delavayi*Franchet (1896: 267) has been discussed, suggesting that the diagnostic value of staminodes could have been overestimated (Suksathan 2009; Wang et al. 2018; Yu et al. 2018).

Since no significant differences were found among these four taxa, we propose reducing *Parnassia tibetana*, P. nubicola subsp. occidentalis, and P. nubicola var. nana to synonyms of *P. nubicola*.

## Taxonomic treatment

### Parnassia nubicola

Taxon classificationPlantaeCelastralesCelastraceae

Wall. ex Royle, 1835: 227

55039D05-3FFB-5325-8A4A-F88E4BCD8F6E

=Parnassia tibetana Z.P. Jien ex T.C. Ku, 1987: 38. Syn. nov. Type: China. Tibet: 2 August 1959, *Exped. Zhufengensis 716* (holotype PE01863973!; isotype N117066161!).
=Parnassia nubicola subsp. occidentalis Schönbeck-Temesy, 1966: 2. Syn. nov. Type: Pakistan. Swat: Utror, 2500 m, 23 August 1962, *Rechinger 19590* (holotype W19680016319!; isotype G00388771!).
=Parnassia nubicola var. nana T.C. Ku, 1991: 82. Syn. nov. Type: China. Yunnan: Deqen, 25 August 1987, *Hengduanshan Exped. 3734* (holotype PE00866291!; isotypes PE00866288!, PE00866289!, PE00866290!).

#### Type.

Nepal. Gossain-than (Gossain-kund), *Wallich Cat. n. 1246a* (lectotype W1889-0305721!, designated by Schönbeck-Temesy 1966: 2; isolectotypes BM000521923!, E00301174!, GZU000100103!, K000739482(except for the individual with fimbriate petals)!).

#### Description.

Perennial herbs, glabrous. Rhizome sympodial, robust. Stems 1 to 5, 5–40 cm, with 1 proximal cauline leaf. Basal leaves 3–8; petiole 1–9 cm; blade ovate or ovate-oblong, 1–9.5 × 0.8–6 cm, base cordate to subtruncate, apex acute or shortly acuminate. Cauline leaf sessile, similar to basal ones but always smaller. Flowers 2.8–3.4 cm in diam., hypanthium campanulate. Sepals ovate to ovate-lanceolate, 4–8 × ca. 3 mm, margins entire, apex obtuse. Petals white, sometimes yellow at the base, obovate to ovate, 8–16 × 5–10 mm, base contracted into a short claw, margins entire distally and erose to short-fimbriate proximally, apex rounded. Anthers ellipsoid, 0.8–1.1 mm; filaments ca. 4.5 mm; staminodes flat, 2.5–5 mm, shallowly to deeply 3-lobed, lobes up to half staminode length, apex acute or rounded. Ovary semi-inferior, ovoid; style ca. 2 mm; stigma 3(–4)-lobed. Capsule ovoid, 5–10 mm long, 3(–4) valved. Seeds minute, oblong, ca. 1 mm long.

#### Chromosome number.

2n =18 (Malla et al. 1979).

#### Flowering and fruiting.

July to November.

#### Habitats.

Margins of thickets, alpine meadows, 2600–4200 m.

#### Distribution.

China, India, Nepal, and also recorded in Afghanistan, Bhutan, and Pakistan (Schönbeck-Temesy 1966, Grierson 1987).

#### Specimens examined.

Herbarium barcode numbers are cited with herbaria acronyms if available.

**China. Tibet, Bomi County**: 11 August 2001, *Qin Haining et al. 337* (PE); 23 August 1983, *Cheng Shuzhi et al. 7062* (PE); 2900 m, 25 August 1975, *Anonymous 56* (SM706501104); 3000 m, 20 September 1973, *Zhang Jingwei 1485* (PE00866285); 3100 m, 20 September 1973, *Tibet Exped. 73-1453* (KUN0437389, KUN0437390, PE00866295, PE00866296); 25 July 1900, *Xia Guangcheng 599* (KUN0437701); **Chayu County**: 3996 m, 29 August 2009, *Gin Xiaohua et al. SET-ET 982* (PE); 3400 m, 9 August 1983, *Tibet Exped. 1334* (QTPMB38503, QTPMB86235); 3350 m, 13 September 1976, *Wu Zhengyi 5833* (KUN0437395, KUN0437398); 3500 m, 13 August 1973, *Anonymous 828* (PE01869747); 3900 m, 13 August 1973, *Tibet Exped. 73-1070* (KUN0437393, KUN0437394); 4100 m, 23 August 1973, *Tibet Exped. 73-1217* (KUN0437391, KUN0437392); **Cuona County**: 10 September 1975, *Tibet Exped. 751933* (PE00866302, QTPMB52545); **Jilong County**: 3977 m, 28 July 2015, *Wei Lai et Hao Jiachen 15338* (BNU0026890); 3544 m, 8 August 2010, *Tibet Exped. 348* (PE01877040); **Jiacha County**: 3500 m, 2 September 1972, *Tibet herbs Exped. 4563* (PE01982462, QTPMB33656, QTPMB82672); **Lang County**: 3239 m, 4 September 2010, *Luo Jian et al. L075* (KUN1237749); without date, *FLPH Tibet Exped. 12-1138* (PE); **Linzhi County**: 3060 m, 26 September 2008, *Gao Lianming et al. GLM-082000* (KUN1242252); 9 August 1983, *Li Bosheng et al. 6375* (PE); 4360 m, 2 August 1975, *Tibet Exped. 751124* (QTPMB51742); **Longzi County**: 10 August 2013, *Chen Yousheng et al. 13-0876* (PE01993054, PE01993055, PE01992603); **Milin County**: 3200 m, 30 July 2016, *Wei Lai et He Yi BNUXZ2016491* (BNU0027963); 3250 m, 21 September 1974, *Tibet Exped. 74-5338* (KUN0437387, KUN0437388, PE00866281, PE01982463); 3700 m, 16 July 1972, *Tibet herbs Exped. 3813* (PE00866276, PE00866277, QTPMB32867, QTPMB82577); **Motuo County**: 2800 m, 12 August 2010, *Jin Xiaohua et al. STET 2646* (PE); 3900 m, 5 September 1982, *Li Bosheng et al. 624* (PE00866297, PE00866298, PE01869745, PE01869746); 3700 m, 2 September 1980, *Plateau Exped. 14709* (PE00866299, PE00866300); **Nielamu County**: 3850 m, 17 July 2016, *Wei Lai et He Yi BNUXZ2016099* (BNU0027962); 3600 m, 2 August 2012, *Mu Xianyun 1174* (BJFC); 3015 m, 3 July 2012, *Gao Lianming et al. GLM-123685* (KUN1241906); 17 August 2011, *Yu Shengxiang et al. 5620* (PE); 18 August 2011, *Yu Shengxiang et al. 5690* (PE); 3970 m, 18 August 2010, *Tibet Exped. 1414* (PE01877046); 1 September 1981, *Ni Zhicheng et al. 1902* (PE); 3400 m, 26 August 1972, *Tibet herbs Exped. 1496* (PE00866274, PE00866275, QTPMB30893, QTPMB80381); 3800 m, 2 September 1972, *Tibet herbs Exped. 1735* (PE00866272, PE00866273, QTPMB31233, QTPMB80851); **Pulan County**: 4500 m, 24 August 1974, *Tibet Exped. 4177* (QTPMB40640); **Yadong County**: 3800 m, 9 August 1975, *Anonymous 75-916* (PE00862011, PE00862012, PE00862013); without detailed locality and date, *Anonymous 464* (P06392577); without detailed locality and date, *Anonymous s.n.* (PE00866303); without detailed locality and date, *Anonymous 465* (P06392739); without detailed locality and date, *Hooker et Thomson s.n.* (P06392739); **Yunnan, Deqin County**: 2900 m, 1 August 1940, *Feng Guomei 5992* (KUN0437384, KUN0437385, KUN0437386); 3400 m, 8 November 1937, *T.T.Yu 7919* (KUN0437382, PE00866269); **Gongshan County**: 2800 m, August 1935, *Wang Qiwu 65371* (PE00866286, PE 0866287); **India. Gangharea**: 15 August 1975, *Anonymous 6550* (PE01982606); **Sikkim**: without detailed date, *Hooker et Thomson s.n.* (P06392774); 22 August 1892, *Gammie s.n.* (P06392778); without detailed locality, 17 August 1849, *Thomson s.n.* (K000739475, K000739476, K000739477, U1467473); without detailed locality, 5 October 1849, *Thomson s.n.* (K000739479, K000739480); without detailed locality and date, *Thomson 145* (K000739482); without detailed locality and date, *Hooker et Thomson s.n.* (P05494790); **Nepal. Kumaon**: 3765 m, 28 August 1995, *F. Miyamoto et al. 9596508* (KUN0579660); without detailed date, *Wallich Cat. n. 1246b* (K000739481, K001112509, K001112511, P06392575, P06392576); without detailed locality and date, *Strachey et Winterbottom 1* (P06392775); **Central Asia.** Without detailed locality, 14 July 1909, *Anonymous s.n.* (P06392740).

## Supplementary Material

XML Treatment for Parnassia nubicola
